# Structures of Oligomeric States of Tau Protein, Amyloid-β, α-Synuclein and Prion Protein Implicated in Alzheimer’s Disease, Parkinson’s Disease and Prionopathies

**DOI:** 10.3390/ijms252313049

**Published:** 2024-12-04

**Authors:** Ondrej Cehlar, Stefana Njemoga, Marian Horvath, Erik Cizmazia, Zuzana Bednarikova, Exequiel E. Barrera

**Affiliations:** 1 Institute of Neuroimmunology, Slovak Academy of Sciences, 84510 Bratislava, Slovakia; 2 Institute of Experimental Physics, Slovak Academy of Sciences, 04001 Kosice, Slovakia; bednarikova@saske.sk; 3 Instituto de Histología y Embriología (IHEM), Consejo Nacional de Investigaciones Científicas y Técnicas (CONICET), CC56, Universidad Nacional de Cuyo, Mendoza M5502JMA, Argentina; ebarrera@mendoza-conicet.gob.ar

**Keywords:** oligomer, amyloid-β, α-synuclein, prion protein, tau protein, neurodegenerative diseases

## Abstract

In this review, we focus on the biophysical and structural aspects of the oligomeric states of physiologically intrinsically disordered proteins and peptides tau, amyloid-β and α-synuclein and partly disordered prion protein and their isolations from animal models and human brains. These protein states may be the most toxic agents in the pathogenesis of Alzheimer’s and Parkinson’s disease. It was shown that oligomers are important players in the aggregation cascade of these proteins. The structural information about these structural states has been provided by methods such as solution and solid-state NMR, cryo-EM, crosslinking mass spectrometry, AFM, TEM, etc., as well as from hybrid structural biology approaches combining experiments with computational modelling and simulations. The reliable structural models of these protein states may provide valuable information for future drug design and therapies.

## 1. Amyloid Aggregation and Oligomer Formation

The aggregation of physiologically intrinsically disordered proteins into insoluble amyloid filaments is a major histopathological feature of many diseases. However, soluble oligomers and not the relatively inert mature filaments are thought to be the toxic agents of several neurodegenerative diseases [1,2,3,4]. The most prevalent neurodegenerative diseases are Alzheimer’s disease (AD) and Parkinson’s disease (PD). The number of dementia cases, out of which AD cases constitute 60–70%, are forecasted to rise from 57.4 million in 2019 to 152.8 million in 2050, according to the Global Burden of Disease Study (GBD) 2019 [5], and the number of patients suffering from Parkinson’s disease is projected to rise from 6.1 million in 2016 to 12.9 million by 2040 [6].

**Figure 1:**
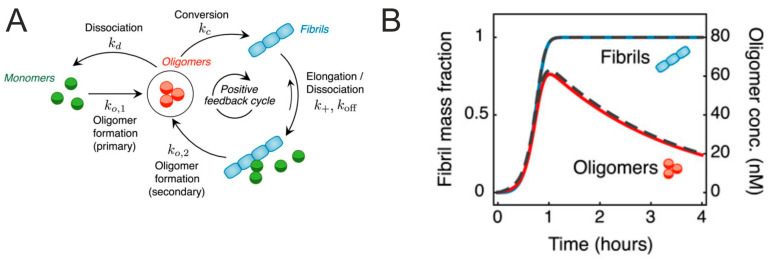
Oligomer formation. (**A**) A schematic representation of the microscopic steps and associated reaction rate constants describing oligomer dynamics during an ongoing amyloid aggregation reaction [8]. (**B**) Kinetics of fibril and oligomer formation. Adapted from ref [8].

The dynamics of oligomer formation have been studied for amyloid-β oligomers and showed that only a minority of oligomers converts into fibrils [7,8]. The key microscopic steps that populate and deplete oligomer populations during amyloid aggregation are shown in Figure 1A. The experimental measurements of fibril and oligomer kinetics are showing a peak of oligomer concentration around the halftime of fibril formation that is followed by a steep decline (Figure 1B).

During amyloid aggregation, monomeric amyloid proteins associate to form dimers, trimers, high-n oligomers, protofibrils and finally fibrils that are deposited in protein plaques (intracellular tau tangles, extracellular Aβ plaques and presynaptic α-synuclein aggregates). Opposite to mature fibrils, oligomers are comparatively smaller (a few nm), mostly globular and characterized by an antiparallel-β sheet structure rather than parallel cross-β strands found in amyloid fibrils [9]. Therefore, oligomers are not sufficiently distinguishable by thioflavin T (ThT) assay [10]. The inter-ring rotation of the ThT molecule is slowed down when bound to amyloid filament, which is increasing fluorescent yields by factors ranging from 160. Therefore, ThT has become one of the most widely used “gold standards” for selectively identifying and analyzing the formation of amyloid fibrils both in vivo and in vitro [10] and the ThT assay is widely used for monitoring of the kinetics of amyloid self-assembly.

Oligomers tend to retain more flexibility in their structure, representing the metastable states among the energy landscape, while fibrils correspond to the ground states of amyloid proteins. On the contrary to fibrillar oligomers, the so-called off-pathway oligomers were shown to inhibit the amyloid fibrilization by depleting the pool of monomers available for fibril nucleation and elongation [11]. However, the role of on- and off-pathway oligomers in the etiology of protein misfolding disease remains controversial. Amyloid fibrillation involves both primary and secondary nucleation [12]. While primary nucleation ensures variability within the oligomers collectively referred to as pre-nucleation clusters, secondary nucleation produces distinct types of filaments [13,14,15]. Amyloid oligomers evolve in distinct arrangements producing annular protofibrils, doughnut-shaped structures, curvilinear protofibrils, spherical oligomers or linear-protofibrils with beads-on-a-string morphology observed by cryo-EM, TEM and AFM.

Amyloid aggregation is a highly sensitive process, governing the structural polymorphisms of filaments, the formation of which depends on many factors, including protein concentration, pH, ion strength, cations, etc. Therefore, explaining the mechanisms of how certain factors modulate the amyloid protein aggregation is of great importance to allow different research groups to prepare morphologically identical amyloid filaments. Under low ionic strength (0–100 mM) conditions, four different types of alpha-synuclein filaments were observed, while at higher ionic strengths, a homogeneous filament sample was obtained [16]. The screening of different reaction conditions and their effect on following tau filament assembly was provided in study conducted by Lovestam et al. [17]. The study revealed that different types of cations, including mono- and bivalent cations present in buffer, led to different protofilament self-assemblies of tau protein, evoking the different disease-specific tau protein folds. The effect of Ca^2+^ on the aggregation pathway of protein S100A9 was monitored to reveal the formation of flexible non-amyloid worm-like protein filaments, while in the absence of Ca^2+^ cations, S100A9 undergoes amyloid aggregation yielding typical amyloid structures [18].

Critical conformational changes to ensure the transition from oligomeric to the first fibrillary form are pending clarification but, considering the structural polymorphism of both oligomeric and fibrillary species, the occurrence of different mechanisms is assumed. Recently, progress in the characterization of protofibrillar intermediate species in the course of the aggregation reaction has been achieved. The study of dGAE Tau (297–391) aggregation by time-resolved cryo-EM showed that disease-specific tau (chronic traumatic encephalopathy—CTE and Alzheimer’s disease—AD) filaments arise from the same filament intermediate (FIA—first intermediate amyloid) during the initial phase of aggregation. The first filament intermediate is composed of two antiparallel tau molecules and already shows characteristics of amyloid cross-packing within an ordered hydrophobic core at residues 305–380, where the tight cross-β interface is composed of tau segment 306–311, which was, interestingly, previously crystallized as amyloid zipper back in 2007 [19,20] (PBD IDs 2ON9, 5K7N). As this interface is not observed in the mature filaments, major conformational rearrangements have to occur in the course of aggregation. After 180 min, various prefibrillar species emerged through FIA-driven secondary nucleation and interface rearrangements leading to the typical C-shaped protofilaments of tau observed in AD and CTE [21], demonstrating that the majority of FIA converts to mature disease-specific filaments. However, the presence of small oligomers in the experiments could not be excluded. Previously, Zhang and colleagues have successfully captured the opening of the α-synuclein annular protofibril ring using cryo-EM and, thus, offered a mechanism of the initiation of annular protofibril aggregation that involves the lateral linkage of two linear protofibrils to produce the bases for filament growth [22]. Recently, intermediate amyloid fibrils of αS from liquid-like spherical condensates have been characterized by ssNMR, cryoEM and biophysical methods to be stabilized by a small core in an antiparallel β-sheet conformation [23].

## 2. Amyloid β

**Figure 2:**
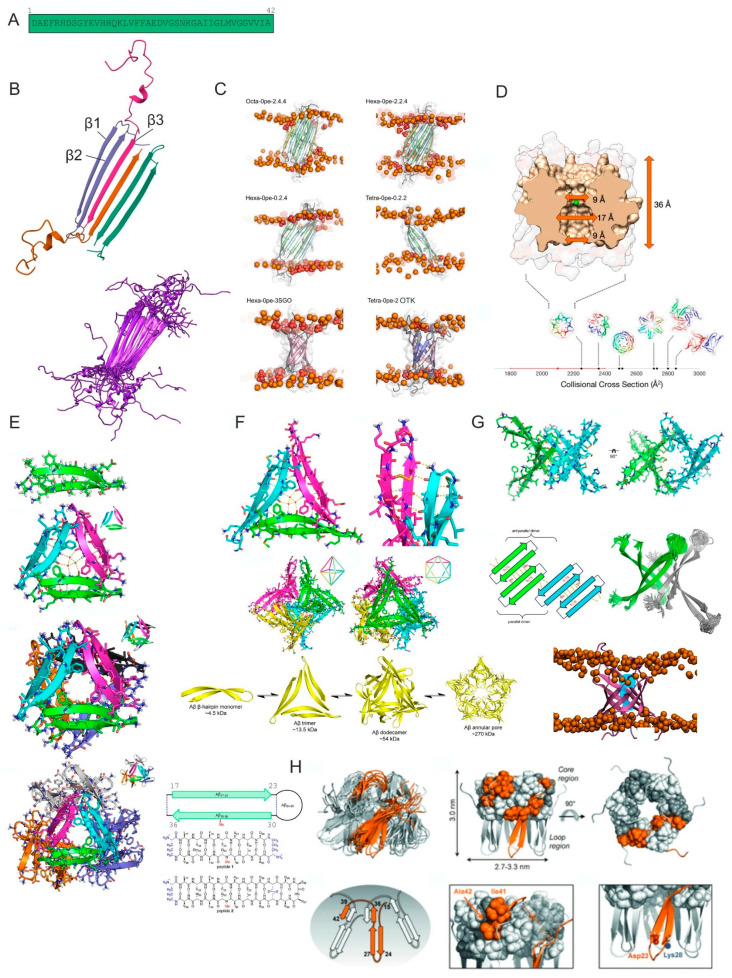
Models of Aβ oligomers. (**A**) Sequence of Aβ1–42. (**B**) The arrangement of different Aβ molecules contributing to the tetramer with β sheets 1–3 shown (**top**); overlay of models from PDB ID 6RHY (**bottom**). (**C**) Representative snapshots of simulations illustrating the extent of the polar defect and conformational deviation for each oligomer model. Models without layered β-sheet edges (0pe = 0 β1-strand pair edges; further numbers indicate the number of β1, β2 and β3 strands)—are shown that do not form stable pores. Bottom row: Models of β barrel pores without exposed edges, based on scaffold structures 3SGO and 2OTK/5W4J respectively [44]. (**D**) Model of Aβ hexameric pore and its collisional cross section (CCS) on the scale of measured values (red line) [45]. (**E**) X-ray crystallographic structure of macrocyclic peptide mimic of Aβ 17–36 reported by Spencer et al. [51]. From top: β-hairpin monomer; trimer, hexamer and dodecamer observed. At the scheme on the right side of the figure, the peptide is assigned as peptide 1. PDB ID 4NTR. (**F**) X-ray crystallographic structure of the trimer reported by Kreutzer et al. [50], formed by macrocyclic peptide mimic of Aβ 17–36. From top: (i) Triangular trimer where the three water molecules in the center hole of the trimer are shown as spheres (**left**) and detailed view of the intermolecular hydrogen bonds formed at the three corners of the triangular trimer (PDB ID 5HOX) (**right**); (ii) views of the dodecamer with octahedral and dodecameric shape; (iii) model for the hierarchical assembly of an Aβ β-hairpin into a trimer, dodecamer and annular pore based on the crystallographic assembly of this peptide mimic. At the scheme on the right side of the panel (**E**), the peptide is assigned as peptide 2. (**G**) X-ray crystallographic structure of the β-barrel-like tetramer formed by peptide mimic of Aβ12–40 (PDB ID 7RTZ): (i) side and top views of the tetramer; (ii) cartoon illustration of the parallel and antiparallel β-sheet interactions that stabilize the tetramer (**left**) and snapshots from REMD simulation of a tetramer of Aβ9–42 based on the β-barrel-like tetramer where residues 12–22 and 30–40 are constrained to the crystallographic coordinates (**right**); (iii) snapshot from the MD simulation showing 24% occupancy of water molecules within the pore over last 400 ns of the trajectory as blue mesh [48]. (**H**) (**Top from the left**) Superposition of the 10 models of Aβcc oligomers with the lowest Rosetta scores: Dimensions of the hexamer barrel with the loop and core regions indicated. (**Bottom from the left**) Simplified representation of the hexamer topology. Packing of C-terminal residues I41 and A42 in the hydrophobic core. Hairpin loop stabilizing D23-K28 salt bridge [54].

Amyloid-β (Aβ) peptide is a deposit of transmembrane amyloid precursor protein (APP), expressed mainly in the synapses of neurons [24]. Its gene is located on the long arm of chromosome 21 [25]. The alternative splicing of APP results in isoforms that range from 365 to 770 amino-acid residues [24]. APP is first cleaved by two types of proteases (α- and β-secretases), leading to either non-amyloidogenic or amyloidogenic processing [26]. The proteolytic products for α-secretase are the soluble APP isoform sAPP-α and the C83 C-terminal fragment, while for β-secretase, these are sAPP-β and C99. Both C-terminal fragments are substrates for γ-secretase, C99 being the precursor of the neurotoxic species [27]. Amyloid-β is normally released throughout human life during synaptic activity and it is indicated to be important for memory and proper cognitive function [28]. In pathological conditions, soluble Amyloid-β has the ability to bind to other Amyloid-β peptides, forming an oligomer and, later, amyloid plaque structures [29]. Although the amyloidogenic process via sequential cleavage by β- and γ-secretases can generate different Aβ isoforms ranging from 37 to 49 amino acids, Aβ40 and Aβ42 (Figure 2A) are the most common forms in the human body. Due to their aggregation-prone nature, studying their monomeric forms results challenging. To cope with this, NMR measurements using different mixtures of hexafluoroisopropanol (HFIP) and water have been conducted, indicating that monomers mostly contain random coil and helical structures [30]. Increasing the water proportion promotes the transition from helical to random coil, ultimately leading to the formation of beta-sheet structures, those observed in pathological aggregates associated with Alzheimer’s disease [31]. Amyloid-β‘s sheet structure contributes to its remarkable stability and resistance to degradation, resulting in its accumulation in the brain [32]. The formation and biological activity of amyloid-β oligomers are central events in the pathogenesis and progression of Alzheimer’s disease [33]. It is also suggested that the size of amyloid oligomers correlates with neural pathology, and smaller oligomers, especially, exhibit more significant toxicity [34].

Experimental pathomechanistic and proof-of-concept studies indicate an imbalance between Aβ neuronal production and the extracellular clearance of Aβ as the upstream event of Aβ dyshomeostasis, associated with protein misfolding, aggregation and incipient extracellular accumulation in plaques [35].

Currently, there are three anti amyloid-β antibodies approved by FDA for treatment of AD (Aducanumab [36,37], Donanemab [38] and Lecanemab [39]), which differ substantially in their binding specificity for different forms of Aβ. Lecanemab is targeting, especially, oligomeric and protofibrillar forms of Aβ, while aducanumab is more specific to fibrils over protofibrils [40] and donanemab is binding the pyroglutamate form of Aβ deposited in amyloid plaques.

### 2.1. Structures of Aβ Oligomers

The assembly of Aβ42 into specific pore-forming β-barrel oligomers—βPFOsAβ42—was observed by Serra-Batiste and colleagues, after they had optimized the dodecylphosphocholine (DPC) micelle conditions. Aβ40, on the other hand, aggregated into amyloid fibrils under these conditions [41]. The Aβ42 oligomers have shown the pore-forming behavior after their addition into the lipid bilayer, as shown by electrical recordings with planar lipid bilayers. This was also observed using membranes excised from HEK293 cells [42].

Later, the group led by Natàlia Carulla resolved the three-dimensional structure of the mentioned βPFOsAβ42 oligomers using solution NMR, IM-MS and XL-MS, which presents the only atomic resolution structure of an oligomer formed by unmodified full-length Aβ42 (Figure 2B) [43]. This model is constituted by six antiparallel β-sheets with two distinct subunits forming Aβ tetramer. Its core is made up of antiparallel β-strands from the Aβ42 C-terminus (β3, residues G29-I41), which is flanking with two β-hairpins, each formed by residues G9-A21 (β1) and G29-V40 (β2). At a higher Aβ concentration, octamers are formed as a β-sandwich of two tetramers. Electrical recordings using planar lipid bilayers and MD simulations have shown pore-like behavior for both tetramers and octamers. MD further showed membrane disruption due to the presence of hydrophilic residues on the edges of both the tetramer and octamer structures, which leads to the reorientation of lipid headgroups that might constitute the molecular basis for the Aβ oligomer toxicity in AD.

Matthes and de Groot have used this oligomer model to construct a pore inserted into the phospholipid bilayer and performed further MD simulations, comparing it also with other pore arrangements (Figure 2C) to establish a principal relationship between the three-dimensional Aβ42 oligomer structure and membrane permeabilization. Their results suggest that membrane-inserted, layered β-sheet edges are a key structural motif in pore-forming Aβ42 oligomers independent of their size [44].

Another group has observed amyloid-β hexamers as the largest observed oligomers in a membrane-mimicking environment using native IM-MS (Figure 2D) (using Carulla’s protocol) [45] and, later, highlighted the importance of hairpin structures for the formation of disease-related oligomers [46].

The observed conformation has underlined the importance of β hairpins proposed earlier by the crystallization of mutated amyloid-β peptides. β-Hairpins are intramolecular antiparallel β-sheets composed of two β-strands connected by a loop or turn and are hydrogen bonded to one another. Three decades of research suggests that Aβ peptides form several different β-hairpin conformations, some of which are building blocks of toxic Aβ oligomers [33]. The first structure of β hairpin formed by Aβ was published by Hoyer and coworkers in the complex with affibody (PDB ID 2OTK) [47]. The group of Prof. Nowick has extensively studied the formation of Aβ oligomers from stabilized β-hairpins [48,49,50,51,52,53]. They have, first, determined the structure of the macrocyclic peptide mimic of Aβ 17–36, in which residues 17–23 (LVFFAED) and 30–36 (AIIGLMV) form the β-strands of a β-hairpin and are covalently linked by two δ-linked ornithine β-turn mimics. An N-methyl group was incorporated at position G33 to prevent the uncontrolled aggregation and precipitation of the peptide and to improve solubility; the residue M35 was replaced by ornithine. The X-ray crystallographic structure of this peptide reveals that it folds to form a β-hairpin that assembles to form trimers and that the trimers further assemble to form hexamers and dodecamers (Figure 2E, PDB 4NTR). REMD simulations were performed, also incorporating residues 24–29 omitted from the crystallized peptide [51]. Later, they designed a homologue peptide that restores the 24–29 loop and M35, but residues V24 and G29 were mutated to cysteine and linked by a disulfide bond (Figure 2F). This peptide assembles hierarchically in the crystal lattice to form trimers, dodecamers and annular pores that resemble in size the annular protofibrils [50]. Recently, they have studied a series of β-hairpin peptides derived from Aβ12–40. Dye-leakage and caspase 3/7 activation assays using tetramer and octamer forming peptides from this series reveal membrane-damaging and apoptotic properties. A 500 ns long molecular dynamics simulation of the β-barrel-like tetramer embedded in a lipid bilayer using NAMD 2.14 and CHARMM36 force field shows membrane disruption and water permeation (Figure 2G, PDB 7RTZ) [48]. Interestingly, these oligomers have the same tetramer and octamer stoichiometry as the βPFOsAβ42 oligomers described by Carulla’s group but are in β-barrel-like conformation. Taken together, the crystallographic structures of oligomers obtained with stabilized β-hairpin forming peptides may provide high-resolution structural information useful for drug and antibody design.

Using solid state NMR, the structure of Aβcc oligomer (with A21C and A30C mutations that arrest Aβ at protofibril stage) composed of hexameric peptide barrel has been described (Figure 2H) [54].

The thermodynamics of α-helix to β-sheet conversion of Aβ1–40 on small unilamellar vesicles has been described. The fitting of the data suggests the formation of octamers [55].

### 2.2. Models of Aβ Oligomers Obtained by MD

**Figure 3:**
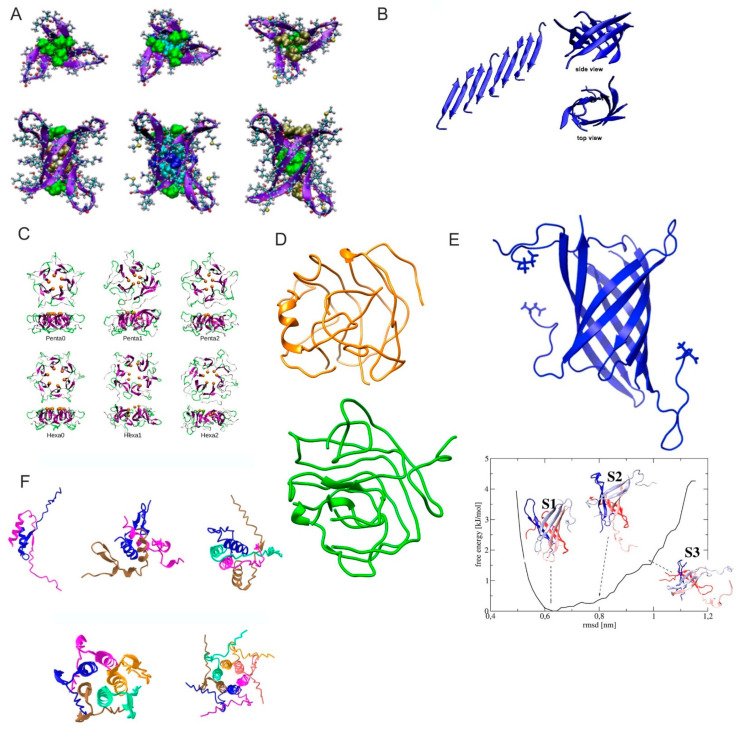
MD obtained Aβ oligomer models. (**A**) Trimers of C-terminal Aβ tandem repeat fragments. Left to right: Aβ(24–34), Aβ(25–35) and Aβ(26–36) GG in cylindrical conformation. Adapted from [56]. (**B**) Initial out-of-register antiparallel starting conformation of Aβ(25–35) and final octamer conformation after MD simulation. Adapted from [56]. (**C**) Top and side views of Aβ42 oligomers generated by Heligeom59 (Penta0 and Hexa0) and by MD simulations (Penta1, Penta2, Hexa1 and Hexa2). The C-terminal residues Ala42 are represented in an orange sphere. Adapted from [58]. (**D**) Two most populated clusters of Aβ42 tetramer. Adapted from [63]. (**E**) MD-refined [69] β-barrel conformation of tetrameric Aβ40 (MD starting conformation) (**top**); free-energy landscape of tetrameric Aβ42 with respect to the top conformation using AMBER99SB-DISP and residues 11–28 and 30–42 (**bottom**). Adapted from [64] with permission from ACS. (**F**) Representative structures of AlphaFold2 predicted structures of Aβ42 oligomers (from dimer to hexamer) showing the helical interface made by the C-terminus. Adapted from [66].

The formation of cylindrin-like structures similar to those previously obtained for αB-crystallin [56] was proposed by Do and coworkers using IM-MS in combination with EM, AFM and computational modeling and Aβ fragments Aβ(24–34), Aβ(25–35) and Aβ(26–36) together with tandem repeats of these peptides connected with GG linker [57]. The IM-MS data reveal the existence of hexamers in the aggregation cascades of all single-stranded Aβ fragments (Figure 3A). MD simulations used the OPLS-AA force field. Single Aβ fragments were shown to form octamers by IM-MS and standard MD simulation (Figure 3B).

Tran and coworkers have performed REMD simulations of Aβ42 with the OPLS-AA force field to characterize its conformational space [58]. The REMD-obtained conformational ensemble was compared with ensembles obtained by NMR measurements by comparing the calculated ensemble-averaged ^3^J_HN-Hα_ coupling constants to the experimental data [59,60]. They have, further, built coarse-grained models for the ring-shaped pentamers and hexamers of Aβ42 with docking calculations using the SCORPION force field and Heligeom module in PTools and their stability was probed with all-atom MD [58,61,62] (Figure 3C).

H.L. Nguyen and coworkers have used a coarse-grained force field UNRES, and REMD methodology to search for the conformational space of the Aβ42 tetramer [63]. They have obtained five clusters out of coarse-grained simulations. (Figure 3D, structures for the two most-populated clusters out of five are shown). The stability of the resulting models was further evaluated using all-atom MD simulations using either the AMBER99SB-ILDN force field with TIP3P or the OPLS-AA/L with TIP4P water model.

P.H. Nguyen and coworkers have performed REMD simulations using four protein force fields—Amber99SB-ILDN/TIP3P, OPLS/TIP3P, CHARMM36m/TIP3P-modified and Amber99SB/DISP—and identified the propensity of Aβ40 and Aβ42 to form tetrameric β-barrel structures in aqueous solution (Figure 3E) [64]. This group has recently published models of Aβ42 oligomers produced by AlphaFold2 [65,66] (Figure 3F). It is worth mentioning the limitations of the chosen methodology when reproducing amyloid structures that are characterized by their low population on structural data bases and high polymorphism. This is why other integrative docking approaches should not be disregarded, like Haddock [67], which incorporates low-resolution experimental data into its conformational search algorithm. The recent release of Alphafold3 [68], with higher intermolecular interaction prediction accuracy, opens a new range of possibilities to model amyloid landscapes.

### 2.3. Structures of Aβ Dimers

Due to its transient and aggregation-prone nature, there is a lack of experimental structural information about Aβ dimeric species. This is one of the cases where the computational microscopy [70] becomes a valuable alternative to obtain atomic-level models. In 2016, Birgit Strodel published a minireview on Amyloid oligomers [71] encompassing publications during the years 2012 and 2015. Regarding Aβ dimers, simulations addressed the driving forces and mechanisms leading to dimerization [72,73]; the effect of the fibrillization inhibitor (−)-epigallocatechin gallate on the cross-correlation sections and secondary structure contents of Aβ1–42 dimers [74]; the additional interactions generated by Thr43 in Aβ1–43, stabilizing a ring-shaped conformer [75] and the different conformational behavior observed for alloforms Aβ1–40 and Aβ1–42 [76]. All these publications are characterized by simulation replicas reaching the nanosecond timescale, reflecting the available computational resources of the period.

In 2021, Phuong Nguyen continued the literature review on these toxic small aggregates [2], including a subsection of extensive MD simulations, all surpassing the microsecond timescale. The bibliographic search on Aβ dimers covered the period between 2015 and 2021, including several studies from the group of Philippe Derreumaux. These all share the use of replica exchange MD techniques to generate equilibrium ensembles of Aβ1–40 dimers [77], to study the effect of A2T and A2V mutants over its aggregation kinetics [78,79] and how the S8C mutation and its subsequent disulfide bond formation, despite inhibiting the formation of large aggregates, still preserves toxicity [80]. Using the same advanced sampling technique, authors compared the structural outcomes of the OPLS-AA, CHARMM22*, AMBER99sb-ildn and AMBERsb14 force fields. Regarding the Aβ1–42 alloform, Lyubchenko and colleagues studied its dimerization process and analyzed the stabilizing forces using pulling MD simulations, comparing the results with AFM experiments [81].

After 2021, the MD community did not continue pursuing the objective of extending trajectory times. Instead, they focused on applying new enhanced sampling methods and force-fields, specifically tailored to reproduce the partially disordered behavior of Aβ. This was the case for B. Strodel and collaborators, who studied the effects of a neuronal membrane mimicking bilayer on the conformational behavior of Aβ1–42 dimers [82]. They used the CHARMM36m force-field and the modified TIP3P water model, which have been validated to generate accurate backbone ensembles for both IDPs and globular proteins [83]. The authors observed, on simulations with aqueous solutions, that Aβ1–42 dimers presented a random coil to β-sheet transition. Conversely, the interaction of Aβ1–42 with the membrane, specifically, with the ganglioside GM1, inhibited this conformational transition, giving a possible explanation for its neuroprotective effects. Hisashi Okumura et al. used the Amber parm99SB force-field and applied the Coulomb replica-permutation method to study the dimerization process for Aβ1–40 and Aβ1–42. This enhanced sampling MD method differs from the traditional REMD in two ways: First, instead of temperatures, Hamiltonian parameters (in this case electrostatics) are the ones exchanged between replicas; and second, different from REMD, the permutations are given between more than two replicas. The authors observed increased interactions between the C-terminal and Arg5 in Aβ1–42; the relevance of this residue on the aggregation properties was further confirmed by studying the aggregation behavior of mutants Arg5Gly and Arg5Glu by thioflavin T fluorescence assays [84]. The group led by Nicklas Österlund [46] combined low-resolution experimental techniques, machine learning algorithms and MD simulations to study the oligomerization process of different variants of Aβ1–40. Initial monomeric conformations were generated with Alphafold2 [85], obtaining characteristic β-hairpins for the WT form but not for a scrambled variant. The oligomerization processes were studied by conventional MD simulations, using the Charmm36m force-field and the TIP3P water model, collision cross sections obtained from ion mobility mass spectrometry and secondary structures from circular dichroism agreed with MD simulations results. This strengthened the conclusions made by the authors about the importance of a β-hairpin formed between the C-terminal and the central hydrophobic core (residues 17–21) in Aβ early aggregation steps.

### 2.4. AFM of Aβ Oligomers

**Figure 4:**
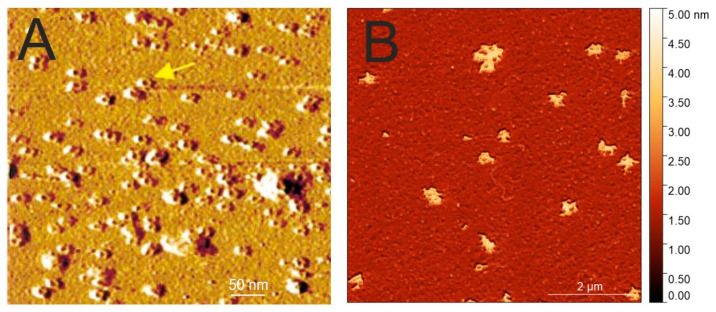
AFM images of Aβ oligomers. (**A**) Ion channel pores constituted by Aβ42. Yellow arrow points to one of the pores. Adapted from [86]. (**B**) AFM image of Aβ1–40 tentative oligomers showing signs of pore-like structure (Z. Bednarikova).

Low-resolution observations of Aβ oligomers can be obtained using atomic force microscopy (AFM). Lin and coworkers have shown ion channel pores constituted by Aβ42 after the incorporation into a planar lipid bilayer (Figure 4A) [86]. They have also shown the formation of globular Aβ42 structures in solution for the period of up to 8 h. We have observed signs of pore-like structures formed by Aβ40 without the addition of lipids (Figure 4B). The interactions of Aβ with membranes have been recently extensively reviewed by Viles [87].

The study using atomic force microscopy–infrared (AFM-IR) spectroscopy has observed the presence of the majority of oligomers with a parallel β-sheet arrangement at the early stage of aggregation (4 h) that exhibited, however, slower rates of fibril formation in comparison to the oligomers with antiparallel β-sheets, which started to appear in the middle stage of the aggregation reaction (24 h) and rapidly propagated into protofilaments and fibrils [88]. This brings an important observation for the structural change in the β-sheet arrangement needed for the progression from oligomers to fibrils.

### 2.5. Isolation of Aβ Oligomers

A study on the effect of soluble amyloid-beta (Aβ) oligomers isolated from the cerebral cortex of patients with Alzheimer’s disease (AD) on synaptic plasticity and memory was published by Shankar and colleagues [89]. When applied to a normal rodent hippocampus, the oligomers inhibited long-term potentiation (LTP), potentiated long-term depression and diminished dendritic spine density. The performance of normal rats in passive avoidance conditioning testing learned behavior was also impaired. Isolation of Aβ was performed by the sequential centrifugation of tissue homogenates and preparation of the following fractions: aqueously soluble (Tris-buffered saline (TBS)), detergent-soluble (TBS with 1% Triton X-100) and ‘insoluble’ (5 M GuHCl). Immunoprecipitation and western blotting discovered Aβ monomers and lithium dodecyl sulfate (LDS)-stable dimers and trimers in all three fractions of specimens from subjects with clinical and histopathological diagnosis of AD. The concentrations of total Aβ, as determined by ELISA, were 1.93 to 2.34 nmol Aβ/g brain tissue in the insoluble extracts and 0.04 to 1.44 pmol Aβ/g brain tissue in the soluble extracts of AD cortex. Since the clinical progression of AD correlates most strongly with the profusion of TBS-soluble Aβ [90], the authors chose to focus on the characterization of the physiological effects of this extract. After immunoprecipitation, GuHCl extracts of Alzheimer’s disease cortices separated on LDS-PAGE gels underwent mass spectrometry to confirm the presence of Aβ dimers. With this method, the authors detected an 8-kDa species. Next, nondenaturing size-exclusion chromatography was used to separate the various Aβ species in the TBS fraction. The Aβ eluted in the void volume (>60 kDa) has dissociated into monomers and dimers after denaturation in LDS-PAGE. Aβ dimers were eluted at ~8–16 kDa and monomers at ~3–6 kDa. Out of these SEC fractions, each tested separately, only the one containing dimers significantly inhibited LTP. Synthetic Aβ 40-S26C peptides were generated to test the hypothesis that ligand binding to Aβ dimers is required for the impairment of LTP. These synthetic dimers, which cannot contain any other factors from AD TBS extract, had a 20-times stronger inhibitory effect on LTP. To test the effect on LTP of insoluble amyloid plaques, which correlate poorly with AD progression [90,91,92], foci of fibrillar Aβ were isolated from neuritic plaques. TBS-insoluble pellets of plaque-rich Alzheimer’s disease cortex were homogenized in 2% SDS. Congo red staining of the residual pellet highlighted intact amyloid. This highly insoluble aggregate was then solubilized with formic acid to release Aβ dimers and monomers. The resulting extract was able to inhibit LTP in hippocampal slices significantly.

Using competitive phage display selection, Morgado and colleagues have generated an antibody fragment, KW1, with conformational specificity able to recognize not only Aβ oligomers but also their different types [93]. The authors used immobilized Aβ (1–40) peptides as baits and, as a competitor, the dissolved, mostly monomeric, peptides of the same type. The resulting antibody fragment was genetically fused with alkaline phosphatase to create a homodimeric protein, KWA1AP. The conformational specificity of the KW1 was confirmed by spot-blot, ELISA and surface plasmon resonance. To test antibody specificity on human samples, Aβ was extracted from the occipital and temporal cortices of an Alzheimer’s disease patient (Braak and Braak IV) and an aged control subject. The tissue was homogenized in Tris-buffered saline and centrifuged. The obtained supernatant was subjected to filtration on a 30-kDa membrane, then retained, and the prepared extract underwent immunoprecipitation with KW1AP. Urea gel electrophoresis revealed the potent binding of antibody fragments to oligomeric Aβ (1–40) species.

In their study of neuropathologically diagnosed Alzheimer’s disease patients, Savioz and colleagues performed neuropathological and biochemical analyses of Aβ oligomers [94]. A total of 21 subjects (10 AD cases and 11 aged controls) with available fresh tissue underwent Western blotting. Two protocols were used for homogenization of the tissue from the temporal cortex and from the cerebellum as a control. Lysis buffer containing protease inhibitors with and without SDS was used. After centrifugation, protein lysate was then separated by PAGE. The protocol without SDS in lysis buffer resulted in a lower concentration of overall proteins. Out of all detected signals, only the band near 55 kDa had a significantly increased signal in AD cases compared to controls. This signal corresponded to a putative 53 kDa-sized dodecamer of Aβ (1–42) [95] or to the putative dodecamer of Aβ*56 [96,97]. The strength of this signal correlated with the density of senile amyloid plaques, but not with the neurofibrillary tangle densities in the temporal cortex.

Another group generated a monoclonal antibody targeting a conformational epitope computationally predicted to represent Aβ oligomers, but not monomers or fibrils, in the hopes of its potential clinical application in the treatment of AD [98]. The resulting antibody PMN310 was raised against the computationally identified sequence HHQK (Aβ residues 13–16), which was replicated in cyclic form and used for mice immunization. The authors performed both in vitro (to test its effect on Aβ oligomer propagation and toxicity), and in vivo studies (to explore its effect on memory formation, synaptic loss and inflammation). Its humanized version (huPMN310) was also tested and its selective binding to Aβ oligomer-enriched brain fractions and lack of adverse-event-associated binding to amyloid deposits compared favorably with other existing antibodies targeting Aβ. huPMN310 was tested on samples obtained from the frontal cortex of 17 AD patients and four controls. Tissues were homogenized in TBS with protease inhibitors and then ultracentrifuged. The resulting pooled soluble brain extracts were then subjected to size-exclusion chromatography. Two fractions—one with HMW—high molecular weight (>140 kDa) and another one, LMW—low molecular weight (~8 kDa to ~70 kDa, ~4.5 kDa-sized Aβ monomers were excluded) were collected. MesoScale analysis revealed total Aβ38, 40 and 42 species to be present in both fractions at similar levels and that, specifically, Aβ42 was overrepresented in aggregated oligomeric Aβ. HMW fractions contained significantly higher levels of both aggregated Aβ42 (43.8 pg vs. 1.3 pg) and aggregated Aβ40 (3.4 pg vs. 0.37 pg) than LMW fractions. After necessary preparation, collected fractions underwent surface plasmon resonance analysis to assess their binding to huPMN310 and other Aβ-directed antibodies. huPMN310 consistently showed high and preferential binding to LMW fraction, compared with aducanumab and bapineuzumab. Authors posit that it corresponds to the preferential recognition of small Aβ oligomers and selectivity of aducanumab to any aggregated Aβ. Then, sandwich SPR with aducanumab as a detector antibody was performed to assess the specific binding to Aβ in LMW fraction. This setup produced a consistently strong signal, both with huPMN310 and aducanumab as a capture antibody. When huPMN310 was used as the detector and PMN310 or aducanumab as capture antibodies, the signal was diminished in comparison to aducanumab detection, in line with Aβ oligomeric binding sites in LMW being already occupied by the capture antibody, since aducanumab mostly captures different Aβ conformations from those detected by PMN310. In the competition assay, pre-exposing huPMN310 to its cognate cyclic peptide epitope blocked its binding to the LMW fraction. The analogous test of aducanumab in LMW fraction had a negligible effect because aducanumab is specific for a different epitope.

Sandberg and colleagues have reported on the Aβ42 oligomer-specific antibody and its effects on the neurotoxicity of AD brain extracts [99]. Antibody ALZ-201 was developed from mice immunized with mutated stable Aβ42 peptide by hybridoma technology. The human brain tissue of AD patients and control subjects was used for post-mortem protein isolation. Samples of temporoparietal grey matter cortices and frontal cortex were homogenized in artificial cerebrospinal fluid and centrifuged and the supernatant was collected; subsequently, they were dialyzed and the MesoScale platform was used to determine the Aβ40 and Aβ42 concentrations. Measured concentrations were present at higher levels in AD brain extracts compared to controls for both Aβ40 (from 67.3 to 393.83 vs. from 29.27 to 47.8 pg/mg protein) and Aβ42 (from 43.32 to 273.75 vs. from 2.88 to 6.15). The levels of oligomeric Aβ could not be quantified by Western blot, likely due to their low amount. In vitro, the application of brain extracts immunodepleted with pan-Aβ antibody and anti-Aβ42 antibody ALZ-201 to mouse primary neurons resulted in a similar effect on the morphological measures of neurotoxicity in high-content microscopy analysis.

## 3. α-Synuclein

**Figure 5:**
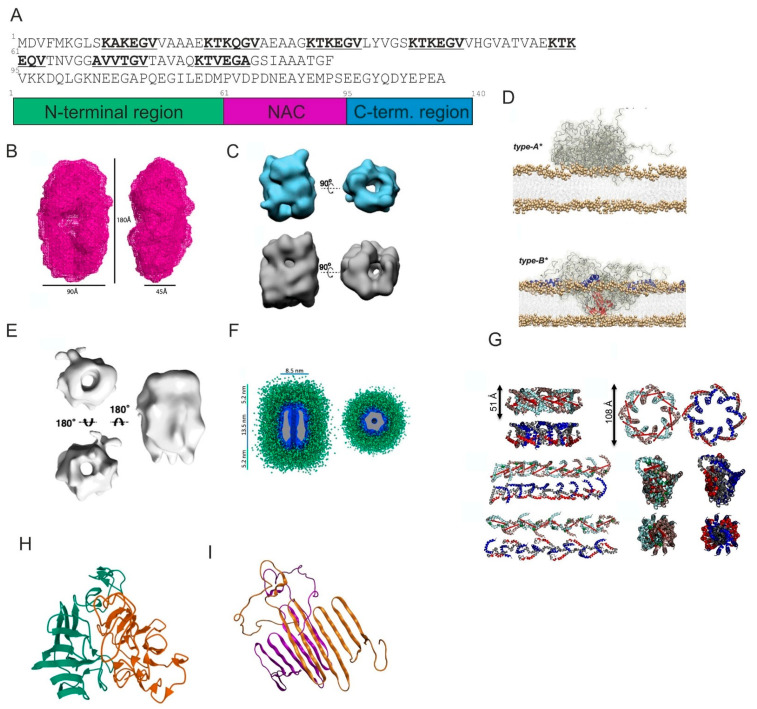
Oligomers of α-synuclein. (**A**) Sequence (3 lines correspond to 3 domains of αS, with 7 imperfect KTKEGV repeats shown bold and underlined) and schema of domain organization of αS. (**B**) SAXS-derived structure of the αS oligomer. Adapted from [112]. (**C**) 10S αS oligomer subgroup (blue) and 15S oligomer subgroup (grey). Adapted from [113]. (**D**) Binding of type A* and type B* oligomers with membranes, where type B* oligomers insert their rigid β-sheet rich regions into the lipid bilayers and therefor disrupt their integrity. Adapted from [114] with permission from the American Association for the Advancement of Science. (**E**) 3D reconstruction of αS oligomers. Adapted from [115]. (**F**) SAXS-based model of αS oligomers showing the compact core in blue surrounded by disordered fuzzy coat shown in green. The cryoEM density map is shown inside the oligomer core (gray). Adapted from [115]. (**G**) Circular (**top row**) and linear/helical/extended (**bottom 2 rows**) oligomeric structures. Adapted from [118]. (**H**) XL-DMD structural model of the αS dimer. Adapted from [119]. (**I**) MD obtained model of α-synuclein dimer, produced by docking of Monte Carlo produced monomers. Adapted from [120] with permission from Elsevier.

α-Synuclein (αS) was first isolated from the electric organ of Torpedo californica in late 1980s and it is a small soluble protein that is highly enriched in the nervous system [100,101]. It is encoded by the SNCA gene on the long arm of chromosome 4 and its full structure consists of 140 amino acids, but alternative splicing in exons 3 and 5 can result in shorter forms (98, 112 and 126 amino acid isoforms) [101]. The α-Synuclein structure includes an N-terminus, a hydrophobic core and an acidic C-terminal tail. The N-terminus, characterized by the presence of repeated KTKEGV sequences in seven imperfect repeats (Figure 5A), typically forms α -helical structures upon interacting with acidic lipid membranes, while the central hydrophobic region (NAC—non amyloidal component) is implicated in the protein’s aggregation-prone nature [102]. The C-terminal domain is variably charged and undergoes significant post-translational modifications, influencing its function and interaction with other proteins [103]. The PTMs of αS contributing to its aggregation include phosphorylation at serine 129 (S129) and other sites, which can either promote or inhibit αS aggregation depending on the context [104]. Ubiquitination generally helps to clear αS and prevent its aggregation, while nitration has been observed to both enhance and inhibit aggregation depending on the specific modified residues [105,106,107].

Functionally, αS is predominantly a neuronal protein, highly expressed in presynaptic terminals of neural tissues. It has been implicated in various essential cellular processes, where αS interacts with synaptic vesicles, proteins (mostly at the presynaptic terminals), lipids, VAMP2, synapsin-Ia, synapsin-III and, also, with itself [108]. Under normal circumstances, αS is a disordered protein, which means that it contains segments that allow this protein to remain flexible and adapt in its folding, exhibiting multiple conformations. These conformations depend on the environment in which it is located. For example, in cytosol, it remains largely unstructured, but upon binding to lipid membranes, it transforms into an α-helical structure. This conformational flexibility is facilitated by the N-terminal domain, which contains lysine-rich repeats that form an amphipathic α-helix, crucial for membrane binding and preventing misfolding [109,110]. Membrane binding is essential for the regulation of neurotransmitter release, as αS affects the assembly of the SNARE (soluble N-ethylmaleimide-sensitive factor attachment protein receptor) complex, which is a critical component for vesicle fusion. αS can bind to SNARE proteins and possibly enhance their assembly and stability, facilitating the approach and attachment of vesicles to the presynaptic membrane [111].

### 3.1. Structures of α-Synuclein Oligomers

Small angle X-ray scattering (SAXS) was used by Giehm and coworkers to construct a low-resolution model of an on-pathway α-synuclein oligomer with maximum distance within the molecule of D_max_ = 180 ± 30 Å. The shape of the oligomer shows that it is an elongated annular species with a central cavity resembling a closed wreath (Figure 5B) [112]. Later, the kinetically trapped α-synuclein oligomers have been characterized by cryoelectron microscopy (Figure 5C). Far-UV circular dichroism (CD) and Fourier transform infrared (FTIR) spectroscopy have shown that the secondary structure content of these oligomers represents the intermediate between that of the monomeric and the fibrillar species [113]. Fusco and coworkers have characterized stabilized type-A* and type-B* oligomers that differ substantially in their ability to disrupt lipid bilayers (type B* were substantially more disruptive) by ssNMR, FRET and AFM (Figure 5D) [114]. Type A* oligomers were prepared by the 48 h incubation of 3 mg/mL monomer with 10 molar equivalents of (−)-epigallocatechin-3-gallate (EGCG) in PBS buffer at 37 °C whereas type B* oligomers were prepared by the 24 h quiescent incubation of 12 mg/mL αS monomer.

Recently, Santos and colleagues have structurally characterized α-synuclein oligomers using ssNMR, SAXS and cryo-EM and their targeting by the peptide phenol-soluble modulin α3 (PSMα3) that is blocking the oligomer-to-fibril conversion (Figure 5E,F) [115]. The peptide binds to N-terminal sequences of αS known to be pivotal for amyloid formation: P1 (36–42) and P2 (45–57) [116,117]. The peptide binding has reduced the oligomer conformational heterogenicity and improved the quality of the 2D classification in cryoEM unveiling the six-fold symmetry.

High-resolution structural information about the membrane-bound oligomeric state of αS was obtained using solution NMR and XL-MS, which help to determine the mode of action of UCB599 phase 2 compound, shown to be interacting with membrane-bound αS oligomers, reducing their number and displacing αS from the membrane [118]. Two oligomeric topologies were observed: circular, ring-like structures and extended, twisted oligomers, models that could be derived by the propagation of two obtained dimer conformations containing α-helical segments (Figure 5G).

Workflow using hetero-isotopic cross-linking constraint guided discrete molecular dynamics has been used to obtain model of α-synuclein dimer, which was chosen out of eight structural subpopulations and was compact, stable and abundant as well as exhibiting partially exposed β-sheet structures (Figure 5H) [119]. Meanwhile, Sahu and coworkers have obtained models of α-Synuclein using molecular docking and MD simulations starting from α-helical and β-sheet rich conformations and suggested that hydrophobic interactions play an important role in the binding affinity (Figure 5I) [120].

### 3.2. Isolation of α-Synuclein Oligomers

The prion-like spread of αS pathology was studied by Watts and colleagues [121] in the brains of mice hemizygous for a mutant A53T α-synuclein transgene [122] inoculated with αS species from the basal ganglia of two patients with the parkinsonian subtype of multiple system atrophy (MSA), a rare disease with prevalence between 3.4 to 7.8 cases per 100,000 [123], and from an aged control. Non-sonicated 1% brain homogenates from humans and homozygous transgenic mice (TgM83^+/+^) were injected into the parietal lobe of weanling mice. Mice inoculated with human brain extracts developed signs of neurodegeneration after approximately 100 days compared with ∼210 for those injected with transgenic mice extracts. Widespread signs of astrocytic gliosis microglial activation and αS hyperphosphorylation were detected in the brains of MSA-inoculated mice. Detergent-insoluble phosphorylated α-synuclein oligomers as well as SDS- and formic acid-extractable phosphorylated α-synuclein, including oligomeric species, were found in the brains of bigenic mice Tg(M83^+/−^:Gfap-luc) inoculated with TgM83^+/+^ or MSA brain homogenates.

The crucial role played by the large oligomeric or protofibrillary αS species in the pathogenic deposition of the protein in synucleinopathies necessitated the development of selective monoclonal antibodies targeted against these species. This need was met by Fagerqvist and colleagues [124], who generated the monoclonal antibodies mAb38F and mAb38E2, highly selective and affinitive for large αS oligomers, able to recognize relevant pathology in the immunohistochemical investigation of synucleinopathic human brains. α-Synuclein was extracted by homogenization in TBS and centrifugation from samples of one cerebral hemisphere and upper spinal cord of transgenic (Thy-1)-h [A30P] [125] α-synuclein mice. The supernatant (TBS-soluble fraction) was collected, and pellets were incubated on the ice in TBS 0.5% Triton X-100. The obtained supernatant (membrane-bound α-synuclein) was stored, and the pellets were resuspended in TBS with 1% SDS. The resulting pellets were resuspended in formic acid and centrifuged and samples were collected. ELISA established the levels of α-Synuclein oligomers/protofibrils in prepared extracts. The levels of oligomers/protofibrils in the spinal cord of 15-month-old mice were 820 ± 915 pM in the TBS fraction, 1265 ± 874 pM in the Triton-X 100 fraction and 2732 ± 5261 pM in the SDS fraction. Analysis of murine brain extracts by sandwich ELISA with mAb38F revealed that the abundance of αS increased with age. Brain extracts from a different subset of transgenic mice were used for subcellular fractionation. These displayed higher oligomeric/protofibrillary α-synuclein levels in the endoplasmic reticulum corresponding to the age of the appearance of behavioral aberrations.

The presence, in the same cell, of both αS and tau in protein aggregates associated with multiple neurodegenerative diseases induced an investigation into the seeding of misfolding of disease-associated proteins. To investigate this interaction, Castillo-Carranza et al. [126] isolated and then purified tau and αS from brain tissues from patients with progressive supranuclear palsy (a rare tauopathy which prevalence ranges from 1.00 to 18 per 100,000 [127]) and Parkinson’s disease (synucleinopathy), respectively. After tissue homogenization and centrifugation, the primary extraction technique used in the study was immunoprecipitation with a T22 (a tau oligomer-specific antibody) for tau oligomers and sequential immunoprecipitation strategy involving T22 and F8H7 (specific for oligomeric αS) for tau oligomers bound to α-S. Finally, isolated complexes were purified by ultra-fast liquid chromatography. AFM was also used to characterize the morphology of extracts in more detail by confirming their oligomeric morphology. The study found that the interaction between the oligomeric αS and tau induces the formation of a hybrid oligomeric species that promotes the misfolding of physiological tau, leading to neurotoxicity and neuronal death, thus exacerbating the pathological mechanisms of neurodegeneration. Furthermore, this interaction might extend the lifespan of tau oligomers and facilitate their spreading in the brain.

## 4. Prion Protein

**Figure 6:**
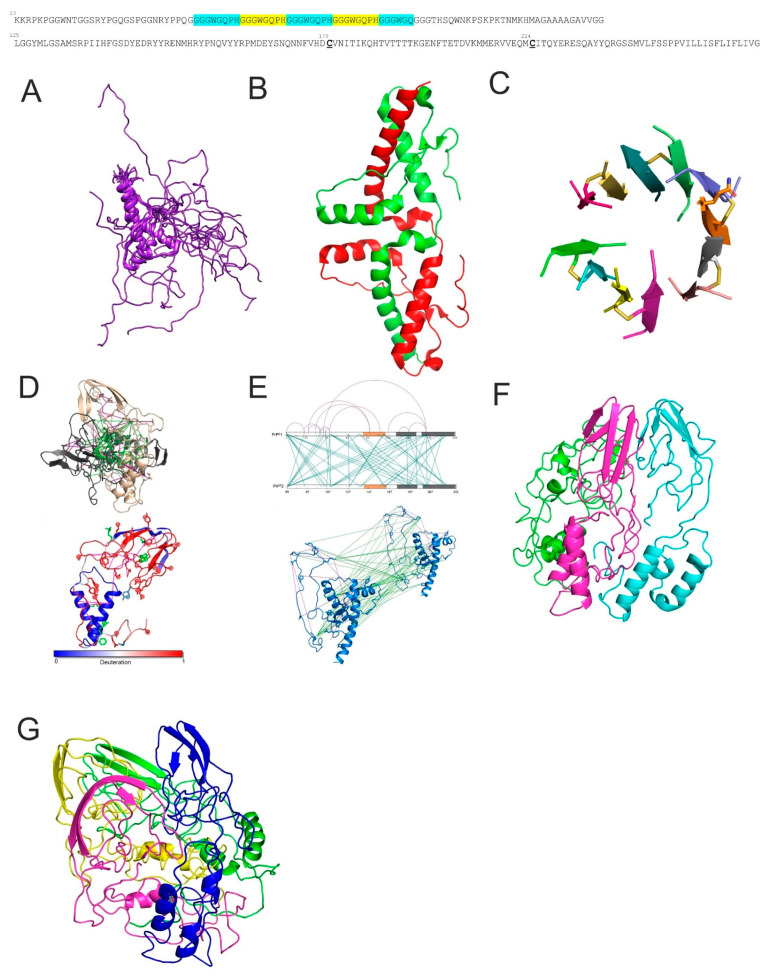
Structure of PrP and its oligomers. (**A**) NMR structure for wildtype human prion protein (PrP residues 91–231) PED00045, PDB 5YJ5, 20 conformers. The sequence of human PrP is shown on top of the figure with highlighted octapeptide repeats and cysteine positions. (**B**) Domain swapped dimer of PrP, PDB ID 1I4M. (**C**) Oligomer composed of PrP peptides, PDB ID 4E1H. (**D**) PrP^β^ dimer structure obtained by short-distance crosslinking constraint-guided discrete molecular dynamics; intra-protein crosslinks (magenta) and inter-protein crosslinks (green) (**top**). Residue deuteration values are superimposed on the representative predicted structure of the PrP^β^ monomer. Intra-protein crosslinks (magenta) (**bottom**). Adapted from [145]. (**E**) Representation of all intra- and inter-protein constraints obtained by crosslinking a 1:1 equimolar mixture of ^14^N- and ^15^N-PrP^β^. Adapted from [145]. (**F**) Model of PrP trimer. (**G**) Model of PrP tetramer.

Cellular prion protein (PrP) is a glycosylphosphatidylinositol-anchored protein, mostly located on the surface of neuron membranes [128]. It is approximately 210 amino-acids long and its structure consists of a flexible tail and structured core. The flexible region consists of the N-terminal domain (residues 23–120), containing an octapeptide repeat region. The structured region is the C-terminal domain (residues 121–231), which is composed of three α-helices and two β-sheets (sequentially arranged β1-α1-β2-α2-α3) and contains two N-glycans (attached at N181 and N197), one disulfide bond and a C-terminal GPI anchor [129]. The NMR-obtained conformational ensemble of human wild type PrP is shown in Figure 6A [130]. Under physiological conditions, prion protein undergoes four cleavages. The α-cleavage in the hydrophobic region of the amino-acid residues (105–120) releases an (~11 kDa) fragment N1. During this cleavage, the 18 kDa part C1 remains attached to the cell membrane by the GPI anchor [131]. The β-cleavage is similar to the α-cleavage and takes place at the end of the octapeptide repeat region. This cleavage occurs approximately at the (residues 90), resulting in fragment N2 (~9 kDa) and fragment C2 (~20 kDa) [132,133]. The γ-cleavage of prion protein was discovered recently, and the cleavage site remains still to be determined. Released fragment N3 (~20 kDa) and GPI-anchored fragment C3 (~5 kDa) indicate that the γ-cleavage occurs between amino acid residues 170 and 200 [134]. The shedding of the prion protein is a very important cleavage, occurring very close to the C-terminus. This cleavage sheds the protein into the extracellular space, resulting in a small amount of amino acid residues on the surface of the cell [135]. Its function involves anterograde and retrograde axonal transport, promoting the myelin maintenance [136]. Prion protein is also indicated to be involved in glutathione reductase activity regulation, acting as antioxidant and via ion binding, regulating superoxide dismutase [137,138]. On the other hand, prion protein has been reported to be involved in neurodegenerative diseases by binding a wide range of β-sheet-rich oligomers, for example, amyloid-β, α-synuclein oligomers and tau aggregates. These toxic oligomers bind to the prion protein at the flexible N-terminal region between amino acid residues (23–27) and (95–110) [139].

Prion proteins are associated with transmissible spongiform encephalopathies (TSE), a group of rare neurodegenerative diseases that are caused by the conversion of a ubiquitous “cellular form” of PrP (PrP^C^) into an aggregated “scrapie form” (PrP^Sc^) [129,140]. Cryo-EM of filaments from extracellular PrP plaques isolated from patients with Gerstmann–Sträussler–Scheinker (GSS) disease, a rare familial disease (incidence 5 per 100 million per year) [141], has been solved, where the filament core spans from residue G80 to residue F141 [142].

### 4.1. Structures of PrP Oligomers

A globular structured C terminal domain of PrP was crystallized in 2001 in a form of a domain swapped dimer with rearranged disulphide bonds and swapped helix α3 (Figure 6B) [143] and the domain swapping was proposed as an important step in the conversion pathway from PrP^C^ to PrP^Sc^. Later, a PrP fragment corresponding to two discontinuous segments derived from α2 and α3 helices of human PrP, ^177^HDCVNI^182^ and ^211^EQMCIT^216^, stabilized by physiologically occurring disulfide-bond between Cys179 and Cys214, was crystallized in the form of hexameric β-sheet rich oligomers (Figure 6C) [144]. The structure of this non-amyloid oligomer is important in the field of prion diseases, because it has been shown that highest infectivity per mass unit is associated with prion particles that are substantially smaller than long fibrils.

XL-MS together with MD simulation has been used to obtain models of dimers, trimers and tetramers of PrP (Figure 6D–G) [145]. The authors have applied urea and mild acid (pH 4) conditions to convert PrP^C^ into PrP^β^ (β sheet rich conformation) [146]. The experimentally obtained short-distance crosslinking constraints were used to guide discrete molecular dynamic simulations (DMD) [147] through which the structure of the PrP monomer and dimer was obtained. The models support the rearrangement and disassembly of the β1-α1-β2 region from the H2-H3 core, which is pivotal to the conversion of PrP^C^ to PrP^β^. The Syrian hamster prion protein encompassing residues 90–232 was used. The obtained structure of the PrP^β^ monomer was further used to obtain models of PrP trimer and tetramer using replica exchange XL-DMD. The proposed β-oligomer assembly provides a clue on the possible β-sheet nucleation site and template-based conversion of the native prion molecules.

### 4.2. Isolation of PrP Oligomers

Chiesa and colleagues [148] focused their attention on molecular mechanisms of the PrP underlying the pathogenesis and transmission of the prion disease. Murine brain homogenates used in the study were prepared by homogenization in PBS and subsequent centrifugation. The obtained lysates were then used in the transmission studies (injection of diluted homogenate into the mouse brain), PK resistance assay, conformation-dependent immunoassay, sedimentation in sucrose gradients and disaggregation of PrP induced by urea. One of the transgenic mice lines used in the study (PG14) expresses a PrP containing a nine-octapeptide insert found in human brains with the familial form of the prion disease (PrP^spon^). Transmission study employing this transgene failed to induce the same fatal neurodegenerative phenotype as the one found in donors, as opposed to PG14^RML^, an infectious and a highly protease resistant form of PrP. Both of these PrP isoforms contain conformationally dependent epitopes in the central and octapeptide repeat regions. The major difference lies in their oligomeric state, namely, the markedly larger size of PG14^RML^ aggregates and their higher resistance to disaggregation in denaturing environment.

Sasaki and colleagues [149] examined the role of oligomeric species in the pathogenesis of a prion disease in a transgenic mouse model with the aim of designing a novel diagnostic tool. As opposed to standard detection methods focused on the detection of pathogenic PrP^RES^ (protease-resistant PrP), the isoform PrP^Sc^ was of particular interest. They examined the formation and evolution of oligomeric PrP deposits in a NZW mouse brain following the inoculation with a mouse-adopted Fukuoka-1 strain of PrP derived from a GSS patient’s brain and created a simplified size-exclusion gel chromatography assay without the need for a proteinase K digestion. Post-inoculation, mice brains were collected biweekly (three at a time) until their death from the disease after three months. These brains, together with sham-operated controls (injected with a normal brain homogenate), were homogenized in extraction buffer with protease inhibitors. The resulting extract was used for PrP^RES^ detection assay involving SDS-PAGE and Western blot. Further analyses included modified spin-column filtration to retrieve proteins with an estimated size ranging from 30 kDa (eluted mainly in fractions 6–8) to >200 kDa (fractions 2 and 3). Fractionated PrP was then examined with PK treatment, Western blot and deglycosylation by PNGase F to gauge the total PrP^Sc^. Dot-blot analysis with an anti-oligomeric antibody was used to detect the presence of oligomers. They managed to clearly separate PrP oligomers from monomers. Interestingly, the ratio of PrP oligomers increased from day 90 post-inoculation, before the increase in the PrP^RES^ fraction from day 105, indicating that this early rise in the share of oligomeric PrP was driven by PrP^Sc^. Furthermore, proteinase K and insolubility in phosphotungstic acid precipitation has shown a rise in the insolubility of PrP oligomers with the disease progression, pointing to the possibility that the distinction between the oligomeric PrP and the physiological PrP^c^ or toxic PrP^RES^ is not absolute, but rather a continuous spectrum.

Xiao’s group [150] attempted to differentiate between iatrogenic (iCJD) and sporadic (sCJD) forms of Creutzfeldt–Jakob disease (CJD) in a comparative study of disease-associated prion protein (PrP^Sc^). CJD is the most common prion disease with worldwide incidence of 1–1.5 cases per million per year [151]. The homogenates of brain tissues of patients with iatrogenic, sporadic and variant CJD were prepared by manual homogenization with a pestle on ice in similar contests as in the paragraph above. The lysate then underwent proteinase K-digestion and deglycosylation. Researchers submitted the PrP lysate to a range of methods—sucrose step gradient sedimentation, conformational stability immunoassay, transmission study, fragment mapping and protein misfolding cyclic amplification (PMCA). Although the amount of PrP was slightly higher in iCJD than in sCJD, most of these methods detected no notable differences in the molecular properties of PrP, except for PMCA and fragment mapping. The seeding activity of PrP^Sc^ was dependent not only on polymorphism but also on the causal mechanism. The amplification efficiency of a sporadic CJDVV2, was significantly lower than the one observed in PrP^Sc^ with the identical polymorphism but of an iatrogenic origin. The authors suggest that this might be explained by prion adaptation driven by conformational alterations that occur during prion transmission [152]. Another distinguishing feature of sCJD cases was the presence of the C-terminal PrP-CTF12/13 fragment in all samples as opposed to iCJD, where it was found only in 25% of the examined brains and none of the three vCJD cases. The authors speculate that this might point to a prion formation pathway distinct for acquired prion disease with PrP^Sc^ formed on the surface and released directly outside, bypassing intracellular processes. They posit that the PrP-CTF12/13 fragment might be formed in the endoplasmic reticulum during a different prion formation pathway associated with spontaneously arising prion disease [153]. However, the study did not find a significant difference in the oligomeric state between iCJD and sCJD using sucrose step gradient sedimentation or gel filtration chromatography. The ratio of PrP in the peak maxima of the oligomeric fractions in gel filtration analysis (<150 kDa) to the total PrP could be quantified to represent 7–8%.

PrP oligomers are also involved in the rapidly progressing AD (rpAD). In their study, Shafiq and colleagues [154] described the interacting partners of high-density oligomers of the prion protein (HDPs) specific for rpAD. For this purpose, protein extracts were isolated from patients with slowly progressing AD (spAD), rpAD, dementia with Lewy bodies, patients with other rapidly progressing dementias including sCJD and controls. Centrifuged homogenates were separated on a 10–45% sucrose gradient. After ultracentrifugation, 20 density fractions were collected and protein–protein interactions were identified with co-immunoprecipitation and further characterized with tandem mass spectrometry. Interestingly, for both rpAD and sCJD samples, HDPs were detected in the same density fractions, pointing to the similarities of their oligomeric density profiles. Due to its crucial role in cytoskeletal regulation disrupted in late-stage AD, growth arrest-specific 2-like 2 protein (G2L2) was selected from interactors for further investigation. Western blot revealed no differences in expression between rapidly and slowly progressing patients. Immunohistochemical analysis revealed that the highest level of G2L2-PrP co-localization was found in rpAD extracts. Confocal laser scanning microscopy revealed disrupted actin–tubulin co-alignment associated with G2L2-PrP co-localization. The association of HDP with cytoskeletal disintegration in rpAD was also confirmed by proteomic profiling with tandem MS. The authors proposed that this might be due to the competitive binding of HDPs to G2L2 that disrupted the interaction between G2L2, EB-1 and tubulin, which is essential for cytoskeletal integrity.

The attachment of PrP^C^ to the cell surface by glycosylphosphatidylinositol (GPI) is prevented by the nonsense mutation Q227X associated with GSS syndrome. Shen and colleagues [155] led an investigation into the involvement of the GPI anchor in the pathogenesis and transmission of the pathogenic isoform PrP^Sc^. The study utilized the brain tissue of a patient with the GSS heterozygous for PrP-Q227X mutation and from a cadaver with sporadic CJD and of a control, in particular, the frontal superior gyrus (GFS2), cerebellar hemisphere (CER), and gyrus rectus (GRU). Human neuroblastoma cell cultures expressing mutated (Q227X or a mutation linked to familial CJD-T183A) and wild-type PrP were also utilized as an experimental system. First, 10% (*w*/*v*) brain tissue homogenates in the lysis buffer were created and treated with protein kinase in order to digest PrP^C^ and extract the resistant PrP^Sc^. For size, density and shape-based separation, sucrose gradient sedimentation was used. There was no conclusive evidence that PrP oligomers were present in the GSS patient’s brain. The presence of PrP in bottom fractions (larger aggregates) during the sucrose gradient analysis and the detection of bands with the 3F4 antibody in Western blotting within the range expected for PrP^Sc^ oligomers (114 to 34 kDa) were strong indications in support of this thesis. Notably, there was an absence of these features in extracts from a non-CJD control brain. PrP^Sc^-Q227X failed to seed PrP^wt^ from control human brains but succeeded at converting the wild-type PrP^C^ from humanized transgenic murine brain tissues.

## 5. Tau Protein

**Figure 7:**
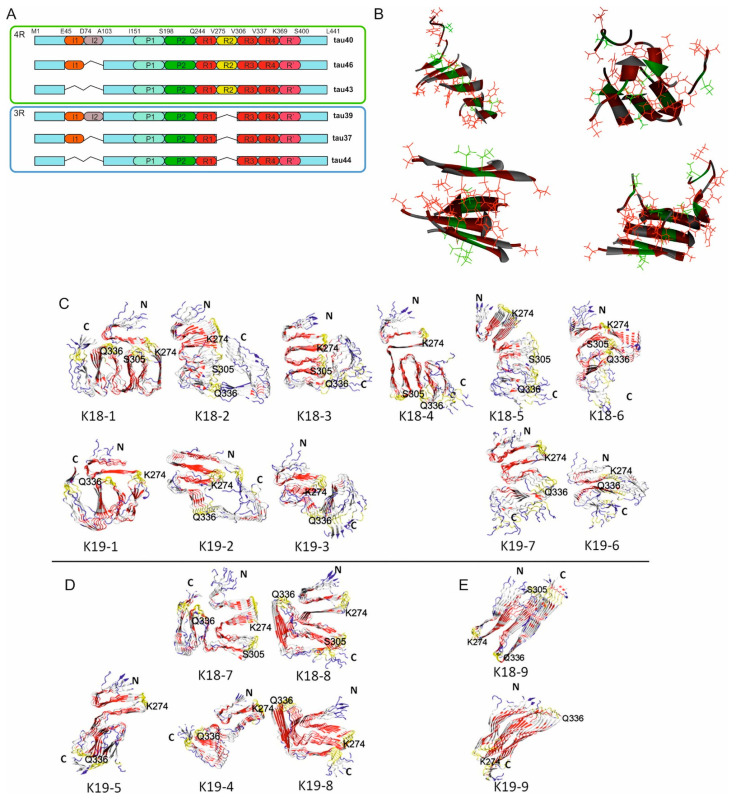
Tau protein and its oligomers. (**A**) Schema of 6 CNS tau isoforms grouped between 4R and 3R isoforms together with their domain organization: I1, I2 represent N-terminal inserts, P1, P2 proline rich regions, R1-R4 microtubule binding repeats (MTBR), R’ region following repeats of lower sequence homology with repeats. (**B**) Snapshots of four meta-stable aggregates seen in the pre-nucleation phase of the 12-chain VQIVYK run at low concentration. V309 residues are shown in green, V306, I308 and Y310 in red. Adapted from [171]. (**C**–**E**) Atomic structures of K18 and K19 octamers, averaged from the last 5-ns MD simulations. Models are divided into three groups according to the conformation of R3 to U-K18/K19 (**C**), L-K18/K19 (**D**) and SL-K18/K19 (**E**) octamers. (**C**–**E**) adapted from [173].

Tau protein is a microtubule-associated protein that is predominantly expressed in neurons, where it plays an important role in the stabilization of microtubules. It was discovered in 1975 and, at first, described as a protein factor that induces tubulin to polymerize into microtubules [156]. It is encoded by the MAPT gene on chromosome 17 and ex-pressed in six isoforms in the human central nervous system, due to alternative splicing of exons 2, 3, and 10. The splicing of these exons leads to the production of isoforms with either three or four microtubule-binding repeats (Figure 7A) [157]. Tau protein has an intrinsically disordered structure and its conformation as well as function might differ in various physiological conditions [158]. Unlike many other proteins, it lacks stable three-dimensional structure and exists in the form of a conformational ensemble [159]. The structure of tau protein is also significantly affected by posttranslational modifications (PTMs) that affect its stability with microtubules and induce its toxic behavior. Tau PTMs include phosphorylation, truncation, acetylation, ubiquitination, methylation, glycosylation, glycation, oxidation, nitration, deamidation and prolyl-isomerization [160]. PTMs also contribute to its aggregation propensity into oligomers and, eventually, into fibrillar forms. These conformational changes have become well associated to a class of neurodegenerative diseases characterized as tauopathies [161]. It is also very important to note the association between tau and cognition. It was observed that the severity of tau pathology correlates with cognitive decline through a variety of mechanisms including, but not limited to, grey matter loss [162]. Similarities between prions and tauons have been proposed [163] after the discovery of prion-like tau transmission [164].

### 5.1. Structures of Tau Protein Oligomers

Oligomers of tau255–441 formed in the absence or presence of heparin were characterized by Peterson and colleagues using PRE (paramagnetic relaxation enhancement) NMR, using four positions of the MTSL spin label [165]. The results have shown signal broadening at regions VQIINK (PHF6*) and VQIVYK (PHF6) after heparin addition for all four label positions. Heparin-induced tau oligomers were later characterized by SAXS to be formed also before the start of the heated and shaken aggregation reaction [166]. Ait-Bouziad and coworkers have characterized stable and toxic tau-phospholipid oligomeric complexes and, using ssNMR and solution NMR, found that tau regions VQIINK and VQIVYK constitute the core of these oligomeric complexes [167]. These experiments are important to elucidate the role of tau-phospholipid and tau-membrane interactions in the pathogenesis of AD.

Recently, super resolution microscopy has shown the presence of tau dimers and trimers on microtubular surfaces ex vivo [168] and tau oligomers were shown to accumulate in the synaptic terminals of AD patients [169].

MD simulations with dimers of peptides containing PHF6* (R2/wt; ^273^GKVQIINKKLDL^284^), its disease-associated mutant ΔK280 or PHF6 (R3/wt; ^306^VQIVYKPVDLSK^317^) have been performed together with IM-MS analyses. Heterodimers containing R3/wt ware less stable than R3/wt homodimers but much more stable than homodimers of R2/wt and R2/ΔK280 [170].

Extensive Monte Carlo simulations with tau fragment AcPHF6 (Ac-VQIVYK-NH_2_) using an implicit solvent all-atom model and of 12, 24 and 36 chains have been performed (Figure 7B) [171] and later described also by Matthes and colleagues, who used 10 copies of PHF6 tau peptide and MD with explicit solvent (GROMOS96 43A1 force field and SPC water model) [172] and showed fast oligomer growth.

Octamers of K18 and K19 (tau variants corresponding to MTBR regions of 4R and 3R tau isoforms, respectively) have been constructed and MD simulations have been performed that have shown the cross-seeding barrier between 3R and 4R tau proteins, where 4R seeds were not able to seed the aggregation of 3R tau (Figure 7C) [173,174].

The research focusing on the aggregation properties of tau PHF6 domain (VQIVYK) is still ongoing [175,176,177,178,179].

The formation of β-barrel like heterooligomers has been shown by REMD simulation of the co-aggregation of tau PHF6/PHF6* and Aβ16–22 peptides [180].

### 5.2. Isolation of Tau Oligomers

Lasagna-Reeves and coworkers [181] discovered that oligomeric tau protein species isolated from AD patients’ brains can propagate their abnormal conformation in endogenous tau of wild-type mice. Successful induction of cerebral amyloidosis and tau pathology was achieved by intracerebral injection of AD brain extracts, containing either pure tau oligomers or PHF tau. In hippocampal slices, the effect of the application on long-term potentiation (LTP) was potently inhibitory and disruptive to memory formation. The authors used three brains of AD patients in a severe stage of the disease and three age-matched controls. Brain tissue was homogenized in PBS with protease inhibitors, lysed on ice and centrifuged, and supernatants (PBS-soluble fraction) were collected. Tau oligomers were isolated from the PBS soluble fraction using immunoprecipitation with T22 antibody.

The seeding and spreading of tau pathology was investigated by Takeda and colleagues [182]. In AD, the progression of tau pathology follows a hierarchical pattern. The authors explored various tau species derived from the brains of mice expressing transgenic tau and AD cases in the neuronal uptake and propagation of pathological tau. Tau species isolated from postmortem cortical extracts and brain interstitial fluid were examined. They discovered the prominent role of endogenous phosphorylated high-molecular-weight (HMW) tau found in low abundance in a PBS-soluble fraction in this process (the main HMW tau size exclusion chromatography fraction represents 4% of the total tau, as determined by ELISA). Tissue from the frontal cortex of four AD patients and three control subjects was homogenized in PBS then sonicated and centrifuged at different speeds (3000× *g*, 10,000× *g*, 50,000× *g* and 150,000× *g*) to separate extract by the size of tau species. This was then confirmed by size-exclusion chromatography. A unique large-pore (1000 kDa cutoff) probe microdialysis technique with a push-pull perfusion system was used to collect HMW tau from brain interstitial fluid (ISF) of awake, freely moving transgenic rTg4510 mice. The collected ISF was then analyzed with SEC followed by human-tau-specific ELISA. The analysis revealed that the ISF also contained HMW tau. Moreover, after a three-day incubation, the murine ISF tau was taken up by primary neurons. This discovery hints at the possible mechanism of tau propagation across the brain.

The generation of conformation-specific single-chain antibody fragments (scFvs) selectively recognizing oligomeric forms of tau has been reported [183]. The biopanning protocol used for their isolation utilized phage-displayed antibody libraries that were then screened by atomic force microscopy. The three antibody fragments produced this way were able to detect tau oligomers in the brain tissue extracts of 3xTG-AD and Tau406+/TauKO (expressing mutant human tau (4R) in a mouse tau knock-out background) mice as well as in the human AD brain. Brain tissue from pathologically diagnosed brains with AD and controls from the middle temporal gyrus (MTG) and mice hippocampi were processed in the following way. Tissue was homogenized in Tris-HCl or EDTA buffer with protease inhibitors and centrifuged and the supernatant was adjusted to a uniform total protein concentration. Samples from human tissues were then used in dot blot assay with an anti-tau-oligomeric scFv.

The group of prof. David Eisenberg [184] reported the generation of a different conformational anti tau oligomer-specific antibody and described its crystal structure and inhibitory properties in regard to disease-related seeding. The monoclonal antibody M204 could specifically recognize tau oligomers induced to aggregate by ionic liquid 15 (50% *w*/*v* 1-n-Butyl-3-methylimidazolium n-octylsulfate). The synthetic single-chain variable fragment (scFv) of M204 was found to form oligomers of distinct molecular weights and the M204-scFv to inhibit pathological tau seeding by extracts from the brains of tauopathy patients. The crystal structure of scFv M204 monomers and oligomers displayed disparate antigen-binding loops pointing to a structural explanation of the amplified inhibitory effects of the oligomeric scFv M204. Tissue from the brains of patients with AD or CTE was sectioned and manually homogenized in 10 mM Tris-HCl Buffer pH 7.4 buffer with 150 mM NaCl and protease inhibitors. Sonicated samples without purification were then employed in seeding experiments. Samples for sarkosyl isolation were homogenized in 10 mM Tris–HCl, pH 7.4 with 0.8 M NaCl, 10% sucrose and protease inhibitors. Following centrifugation, the supernatant was incubated with sarkosyl. Finally, pellets containing fibrils from ultracentrifugation were resuspended in PBS.

A study by Dujardin et al. [185] investigated the reflection of the molecular diversity of tau protein in the clinical presentation of Alzheimer’s disease. The authors have found support for the hypothesis that different properties of the tau protein between individuals influence the spreading of tau pathology through the brain. A wide spectrum of methods was employed including in vitro and in vivo seeding assays, immunohistochemistry, stereology, AlphaLISA, SDD-AGE (semi-denaturing detergent agarose gel electrophoresis), LC-MS/MS, SEC, Proteinase K resistance assay, tau immunodepletion, SPR and whole-exome sequencing. A total of 32 brains of patients clinically and pathologically diagnosed with AD were used. Brains were dissected into hemispheres, with one hemisphere postfixed in 10% formalin for histological studies, while the other hemisphere was coronally sliced at the time of autopsy. Frontal cortex tissue (Brodmann area 8/9) was preserved for homogenization. Frozen tissue was put on wet ice and after thawing manually homogenized in PBS and protease inhibitors and centrifuged. Total tau concentration in the supernatant was determined by a BCA assay. To obtain seeding tau species for in vivo experiments, the PBS-soluble fraction contained in the supernatant was ultracentrifuged and the pellets were resuspended in PBS. The investigation into the tau oligomeric state yielded intriguing results. The authors found that the amount of oligomeric tau was positively correlated with both tau seeding activity and the clinical progression of the disease. Also, most of the time, total tau in brain extracts tracked with monomeric tau. Although HMW fractions had low levels of total tau in general, for individuals with high seeding activity, these levels were increased. Furthermore, enhanced seeding activity and negative clinical prognosis were associated with certain tau phosphorylation sites, notably, at Thr231 and Ser235, and on Ser262. The seeding potential of oligomeric tau might be influenced by an observed variation in the protease sensitivity of tau species from different patients. Thus, tau oligomers might be able to take up distinct conformations impacting their seeding potential. These findings hint at distinct AD subtypes with varying phosphorylation patterns and rates of progression,

Mate de Gerando and colleagues [186] compared the in vivo spreading and seeding properties of fibrillar and oligomeric tau species isolated from the brains of sex-matched patients with AD. Brains of clinically diagnosed, pathologically confirmed AD patients with minimal comorbidities and considered as “high seeders” [185] were selected from the brain bank for protein extraction. First, 5 g of frontal cortex tissue was dissected from Brodmann areas 7 and 8/9. For HMW tau extraction, tissue was dissected as above and homogenized in five volumes of PBS with protease inhibitors in a glass homogenizer on ice. The homogenate was centrifuged at 10,000× *g* for 10 min. The collected supernatant was analyzed by SEC. Fractions containing 400–600 kDa (HMW) tau were pooled and ultracentrifuged at 150,000× *g* for 30 min. Finally, pellets were resuspended in PBS. To confirm the tau-specificity of protein extracts, immunodepletion with an antibody detecting all six isoforms of tau was performed and verified by tau seeding assays and Western blot. The authors discovered that while both SARK (sarkosyl insoluble) and HMW tau have had comparable seeding activity in vitro and in vivo and promote similar temporal and spatial propagation patterns after injection in mouse brains, and that and the effects of both preparations are driven by tau, SARK and HMW tau extracts differ in their tau compositions—with HMW having more oligomeric and SARK more fibrillary tau species—and also diverge in the immune response to the injection, with microglial inflammation clearly identified in the case of HMW tau. The total tau amount in SARK and HMW fractions was quantified by both denaturing WB and non-denaturing ELISA, demonstrating that there is more fibrillar tau per tissue weight than there is soluble HMW species.

In their reply, Stern and Selkoe [187] argued that the method for the isolation of the HMW tau fraction did not enrich the soluble oligomeric tau species, but the biological activity of this fraction could be ascribed to PHF that were still present there as indicated by EM. Furthermore, the authors disputed the downstream characterization of the HMW tau extract with proteinase K, Western blot and ELISA

The former group responded [188] to this by highlighting the characterization of the HMW fraction in the original publication [182] involving detection by oligomer-specific anti-tau antibody that disappeared after denaturation with urea and atomic force microscopy, which demonstrated the structural features of the oligomeric tau. Mass spectrometry revealed differences between HMW and SARK fractions in post-translational modifications [185,189]. They pointed to further differences between tau fractions at the biochemical level as shown by Western blot. The authors also defended and clarified their SEC methodology and described the marginal role of fibrillar tau in the HMW fraction in driving its biological effects and provided a brief overview of the literature supporting their interpretation of findings.

## 6. Conclusions and Outlook

Oligomeric forms of amyloid-β, α-synuclein, prion protein and tau are thought to be the most toxic agents in the pathogenesis of the brain amyloid diseases, but their structural investigation is hindered by their heterogenous and unstable nature. However, the relevance of these oligomeric states is supported by the examples of their isolation from animal and human brain tissues and successive characterization, where specific conformational antibodies play an important role. The structural research focusing on oligomers has brought potential for the design of novel therapeutics, as was evidenced in the case of α-synuclein, and molecules targeting oligomers of remaining proteins can emerge in the future.

The most prominent and recent discussed structural results are for the amyloid-β the structure of βPFOsAβ42 oligomers with a shown pathological effect driven by the edge conductivity in the lipid bilayer complemented by the X-ray structures of stabilized β-hairpin mimicking peptides, and the structure of β-sheet rich oligomers of α-synuclein with hollow cylinder morphology prepared in vitro in solution opposed by the α-helical oligomers formed at the membrane surface. For the PrP and tau protein, there is not a comparable number of oligomeric structural models as for Aβ and αS. This lack of oligomer models for tau protein that needs to be met is probably caused by its higher complexity inherent to its bigger size, the presence of multiple isoforms, truncated forms and posttranslational modifications.

We envisage the emergence of new models for the oligomeric states of the discussed proteins due to the advances in methodologies like the use of high-field NMR (1.2 GHz spectrometers) and high-pressure NMR [190]. Further, the combination of machine learning based structure predictors with the experimental data constitutes a great future promise. Experimental data can be used to select a predicted model with the correct orientation of monomer units. The generation of more specific antibodies or advanced separation protocols can aid in the obtainment of more homogenous oligomeric preparations amenable for the collection of high-resolution cryoEM data.

## Abbreviations

AFM	atomic force microscopy
IM-MS	ion mobility mass spectrometry
REMD	replica exchange molecular dynamics
SAXS	small angle X-ray scattering
SEC	size exclusion chromatography
ssNMR	solid state NMR spectroscopy
TEM	transmission electron microscopy
XL-MS	cross linking mass spectrometry
XL-DMD	cross-linking constraints guided discrete molecular dynamics
